# Healthy Middle-Aged Adults Have Preserved Mnemonic Discrimination and Integration, While Showing No Detectable Memory Benefits

**DOI:** 10.3389/fpsyg.2021.797387

**Published:** 2022-01-24

**Authors:** George Samrani, Anders Lundquist, Sara Pudas

**Affiliations:** ^1^Department of Integrative Medical Biology, Umeå University, Umeå, Sweden; ^2^Umeå center for Functional Brain Imaging, Umeå University, Umeå, Sweden; ^3^Department of Statistics, USBE, Umeå University, Umeå, Sweden

**Keywords:** healthy aging, midlife, episodic memory, memory integration, memory discrimination

## Abstract

Declarative memory abilities change across adulthood. Semantic memory and autobiographic episodic knowledge can remain stable or even increase from mid- to late adulthood, while episodic memory abilities decline in later adulthood. Although it is well known that prior knowledge influences new learning, it is unclear whether the experiential growth of knowledge and memory traces across the lifespan may drive favorable adaptations in some basic memory processes. We hypothesized that an increased reliance on memory integration may be an adaptive mechanism to handle increased interference from accumulating memory traces and knowledge across adulthood. In turn, this may confer an improved ability for integration, observable in middle-age, before the onset of major aging-related declines. We further tested whether the hypothesized increase would be associated with previously observed reductions in memory discrimination performance in midlife. Data from a sample of healthy middle-aged (40–50 years, *n* = 40) and younger adults (20–28 years, *n* = 41) did not support the hypothesis of improved integration, as assessed by an associative inference paradigm. Instead, age-equivalent performance on both integration and discrimination measures were observed [Bayes factors (BFs)_10_ = 0.19–0.25], along with expected higher verbal knowledge and slower perceptual speed for middle-aged [(BFs)_10_ = 8.52–73.52]. The results contribute to an increased understanding of memory processing in midlife, an understudied portion of the lifespan, and suggest that two core episodic memory processes, integration and discrimination, can be maintained in healthy middle-aged adults.

## Introduction

Different facets of our memory processes change throughout adulthood. It is generally agreed that our semantic memory is stable or even increases from mid- to late adulthood, while episodic memory encoding and retrieval begins to deteriorate in late adulthood (Park et al., 2002; Rönnlund et al., 2005; Schaie, 2005). It is also known that previously stored knowledge influences the way our brains encode and store new information (Bartlett, 1932; Craik and Lockhart, 1972; Alba and Hasher, 1983; Anderson, 1984; for reviews see van Kesteren et al., 2016; Gilboa and Marlatte, 2017), but relatively little attention has been given to if, or how, the experiential growth of knowledge and stored memories across the lifespan may influence basic memory processes. Such an information accumulation account of cognitive aging (Ramscar et al., 2014; see also Qiu and Johns, 2020) could in theory entail differential effects on memory processes, where past information can either be beneficial or detrimental during everyday cognition (Umanath and Marsh, 2014). For instance, it has been shown that growth of linguistic knowledge through the lifespan may interfere with new learning of arbitrary word-pairs (Qiu and Johns, 2020) and computational modeling evidence suggests that some apparent age-related declines in recognition memory of verbal stimuli may in fact be explained by increased experience with linguistic stimuli (Buchler and Reder, 2007). Older adults can also draw on specific world knowledge to support memory performance when the nature of the memory task allows, although rarely sufficient to outperform younger adults (Naveh-Benjamin, 2000; Castel, 2005; Umanath and Marsh, 2014; Ryan et al., 2020). The present study explored the possibility of more generalized, task-independent beneficial effects on certain memory processes across the lifespan, as an adaptation to the accumulation of general world knowledge and autobiographical episodic memories across life. By targeting healthy middle-aged adults, we hoped to maximize the chances of capturing beneficial effects of accumulated memory traces and semantic knowledge, while minimizing the risk of potential contamination of adverse aging-related neurodegenerative changes on memory processing known to take place at later ages (Park et al., 2002; Schaie, 2005; Gorbach et al., 2017).

The midlife period, typically defined as ages 40 to ca. 60–65 years, has been relatively understudied in cognitive research (but see Lachman, 2004; Willis et al., 2010; Zimprich and Mascherek, 2010) and few studies have considered potential beneficial cognitive developments that may arise during this life period. Midlife may even mark the peak for some abilities, such as financial decision making (Samanez-Larkin, 2013). Evidence for general episodic memory decrements in midlife, however, is inconclusive so far. While cross-sectional studies have observed linear memory declines from age 20 onward (Park et al., 2002; Hartshorne and Germine, 2015; Henson et al., 2016), some longitudinal studies have found support for later onset of decline, around age 60–65 (Rönnlund et al., 2005; Schaie, 2005). Longitudinal evidence is however also mixed. Modest declines in episodic memory performance starting around age 45–50 were observed in one study (Singh-Manoux et al., 2012), while another study observed significant declines in individuals aged 50–60 at baseline, but not in those aged 40–50 (Hughes et al., 2018). Some of the mixed findings can likely be explained by differences in sample health characteristics, as midlife marks the onset of various health disorders such as hypertension, carotid stenosis, obesity, and type 2 diabetes, all of which can have a negative influence on neurocognitive function (Holden et al., 2012; Ryan et al., 2012; Singh-Manoux et al., 2012; Reagh et al., 2014; Zhao et al., 2015; Hartanto and Yong, 2018). The type of memory process under investigation may also influence the results. For instance, context and source memory tests show robust age-effects cross-sectionally even in healthy middle-aged individuals carefully screened for common health disorders (Kwon et al., 2016; Cansino et al., 2018), which could be indicative of a specific vulnerability of associative memory binding with age (Bender et al., 2010; Chastelaine et al., 2016; Henson et al., 2016; Naveh-Benjamin and Mayr, 2018). Some evidence also exists that memory discrimination, i.e., the ability to discriminate between similar but distinct memory traces is compromised already in midlife (Stark et al., 2013; Nauer et al., 2020; Güsten et al., 2021). In sum, conclusive evidence for a general episodic memory deficit in midlife is lacking, and influence of factors such as sample health characteristics and performance differences across memory sub-processes remain to be elucidated. Thus, the possibility remains that beneficial memory-processing changes may be observed in healthy middle-aged adults, when compared to younger adults.

This study will focus on memory integration, an important sub-process or operation underlying episodic memory. Memory integration is a process by which temporally disparate, related or partially overlapping episodes are thought to become interconnected and potentially stored as overlapping neural representations (Schlichting and Preston, 2015; Backus et al., 2016; Koster et al., 2018). For instance, if one observes a woman entering a house on Monday, and a man entering the same house on Tuesday, the memory traces for this woman and man may become linked. This characteristic of memory has also been referred to as generalization (Kumaran and McClelland, 2012) and inferential memory (Cox et al., 2021)^1^. As such, memory integration supports flexible extraction of new information from memory (Zeithamova et al., 2012; Schlichting et al., 2014), and may underlie the formation of generalized knowledge (van Kesteren et al., 2016), and could therefore be related to semantic knowledge. Importantly, previous research indicates that some forms of memory integration can also reduce memory interference and competition, and thereby increase retention and prevent forgetting (Horton and Wiley, 1967; Moeser, 1977; Anderson and McCulloch, 1999; Konkle et al., 2010; Chanales et al., 2019). For instance, Anderson and McCulloch (1999) found reductions in retrieval-induced forgetting when interconnections were formed across list items during encoding, whether spontaneously adopted or instructed, whereas Postman (1964) observed that conditions that promoted integration across overlapping items in sequentially learned paired-associate lists benefited learning and reduced interference. Integration has further been suggested as a mechanism to avoid the trade-off between encoding and retrieval states, predicted by computational modeling (O’Reilly and McClelland, 1994), that would lead to conflicts between encoding and retrieval processes whenever current experience overlaps with past memories (Richter et al., 2016). This study proceeds from the information accumulation perspective of cognitive aging (see Qiu and Johns, 2020), and conjectures that the increased number of stored memory traces across life may lead to increased interference between similar stored traces (cf., Konkle et al., 2010), as well as more overlap between stored information and new information to be encoded, i.e., proactive interference. We hypothesize that this increased interference may drive an adaptive shift toward higher reliance on memory integration across the lifespan, as one route to reducing interference-related forgetting caused by an accumulation of memories across life. Thus, we reason that the absence of debilitating memory impairment due to interference-related forgetting in midlife, despite substantial accumulation of memory traces, may be accomplished or facilitated by increased integration, which in turn may be observable as a higher performance on test of memory integration in middle-aged adults compared to young. This is the main idea that the current study set out to test. As some previous studies have indicated a potential trade-off between memory integration and the discrimination of details from integrated memory traces (Sweegers and Talamini, 2014; van Kesteren et al., 2016; Tompary and Davachi, 2017), we further wanted to test whether previously observed age-related reductions in memory discrimination in midlife (Stark et al., 2013; Nauer et al., 2020; Güsten et al., 2021) are associated with increased reliance on integrative memory processing in midlife. To our knowledge, memory integration has not been specifically studied in midlife before, although older adults above age 60 have been shown to demonstrate impairment when compared to young (Carpenter and Schacter, 2018). Furthermore, substantial individual differences in integration performance and use of integrative encoding strategies can be found even within samples of younger adults (Anderson and McCulloch, 1999; Schlichting et al., 2014; Varga et al., 2019; Yu et al., 2020).

To explore the hypotheses outlined above, a sample of middle-aged adults (40–50 years), screened for common health-conditions with negative impact on cognitive abilities, was compared to healthy young adults (20–28 years) on commonly used tests of integration and discrimination administered through an online web platform. To capture memory integration, the Associative Inference Paradigm (Preston et al., 2004; Schlichting and Preston, 2015) was used, in which participants separately study overlapping paired associates that share an item (e.g., A-B, B-C) and are thereafter tested on the unseen associative pair (e.g., A-C). For memory discrimination, an adapted version of the Mnemonic Similarity Task (MST) was administered (Stark et al., 2013), which assesses the ability to discriminate between highly similar items from memory. According to the primary hypothesis it was predicted that middle-aged would show higher integration performance than younger adults. The primary measure of integration was scaled for direct associative memory performance, to better isolate integration from explicit memory for the underlying directly studied stimulus pairs. In addition, verbal knowledge tests were administered to verify enhanced semantic memory in midlife, an important tenet of the information accumulation perspective of cognitive aging. The results were expected to not only shed light on important memory processes during an understudied part of the lifespan, but also have the potential to demonstrate adaptive beneficial plasticity in core cognitive functions across adulthood, which in turn would increase our understanding of the backdrop for age-related impairments that arise at later ages.

## Materials and Methods

### Participants

Forty-five younger adults (ages 20–28 years) and 44 middle-aged adults (ages 40–50 years) were recruited nationally from Sweden through flyers, online ads, and by word to mouth. Potential candidates were initially screened by phone interviews in order to recruit a healthy population, and to ensure that participants had suitable equipment to perform the online cognitive testing (see below). Exclusion criteria were conditions and medical treatments that can affect cognitive performance, such as history of brain trauma or stroke, neurological disorders (e.g., dementia, Parkinson’s disease, Multiple Sclerosis), intellectual disabilities, psychiatric disorders, cardiovascular conditions such as heart attacks or hypertension, diabetes, or ongoing cancers. A total of 31 people were screened out prior to testing (see Supplementary Table 1). Of the recruited participants, five were excluded after testing, based on self-reported information from the questionnaires (*n* = 1 for high blood pressure, *n* = 2 for using psychotropic medication, *n* = 2 for refusing to disclose essential information), and another three participants were excluded for not following instructions during testing (e.g., test environment). The final sample comprised 41 younger adults (22 women; mean age = 25 years, SD = 2.1; mean years of education = 15.5 years, SD = 2.1) and 40 middle-aged adults (19 women; mean age = 44.6 years, SD = 3.2; mean years of education = 16.6 years, SD = 3.2). All participants were fluent in the Swedish language and were reimbursed (200 SEK) for their participation. The study was approved by the Swedish Ethical Review Authority. Informed consent was provided verbally by all participants, and documented through an audio recording.

### Testing Procedure

Cognitive testing was done online, with the test leader present through a video call throughout the test session to provide instructions, ensure compliance, and answer questions. During an on-going task, the test leader was muted and did not intervene. As participants were tested on their own computer, we ensured that each participant used a functioning computer with at least a 13″ screen, and a stable internet connection. Cognitive tasks were implemented in a web-based task tool, Pavlovia^2^, with exception for the word fluency task for which participants’ answers were recorded through an audio recording. Surveys including health questions and demographics were sent by mail for participants to fill in and return.

Each task was preceded by an instruction and in some cases a visual presentation of what to expect during the upcoming task. Both the associative inference task and the mnemonic similarity task were introduced using a short 3-min PowerPoint presentation with examples of the upcoming task content and order. The remaining cognitive tasks were explained verbally before each task and with in-task instructions. The cognitive tasks were administered in the following order, from first to last: Mnemonic similarity task, vocabulary, Associative inference task (encoding phase), perceptual speed, Associative inference task (retrieval phase), and lastly verbal fluency.

### Cognitive Tests

#### Associative Inference Task

The AIT task was separated into an encoding phase where participants memorized pairs of images of scenes and faces, and delayed retrieval phases comprising associative inference (indirect) retrieval as well as retrieval of the direct pairs. The associative inference retrieval condition required participants to connect two images that had been previously seen together with the same face, albeit in separate encoding trials. The stimuli set consisted of 92 open access scenery photographs (e.g., buildings, landscapes, famous landmarks) from online websites and 46 color images of human faces (DeBruine and Jones, 2017), see Supplementary Figure 1A. Scenes were categorized into two groups, A and C, while faces made up the B group. The encoding phase consisted of 46 A-B image pairs and 46 B-C pairs, presented in a pseudorandomized order. Every face (B) was in total paired with two different scenes (A, C). Each face-scene pair was presented separately for 3.5 s with an ISI of 1 s, for two repeated encoding sessions separated by a 7 s pause. Participants were told to indicate during each pair presentation with a keyboard button press (down arrow) that they had seen the pair, and attempted to memorize it. Instructions were given to try to visualize each face with the corresponding scene in a relatable self-generated story, with aim to minimize different strategies (or none) to encode new associations. In order to maximize chances of capturing spontaneous tendencies for memory integration, the participants were not made aware that they would be tested on indirect associations before the encoding session.

Participants performed the perceptual speed task (described below) for approximately 7 min before the retrieval phase of the AIT, in order to increase the retention interval. The associative inference retrieval condition consisted of 27 A-C true scene-scene pairs, and 19 reshuffled lure scene pairs from the encoding phase that did not have an overlapping face stimulus. The items were shown in a pseudo-randomized order. For each pair, the participant had to answer on a keyboard either left arrow for “Yes” (“Yes, these scenes are connected to the same face”), or right arrow for “No” (“No, these scenes are not connected to the same face”). The pairs were presented for 5 s, with an ISI of 1 s. A key-response immediately triggered a delay for 2 s before the ISI and the next trial continued. Absence of a response would activate a timeout after 5 s and was counted as an incorrect answer. The direct retrieval condition consisted of 19 A-B and 21 B-C direct face-scene pairs presented in the same manner as the indirect pairs, with 20 incorrect face-scene combinations interwoven in the list. Similarly to the indirect pairs, the participants had to answer on a keyboard either left arrow for “Yes” (“Yes, this face and scene combination was presented previously”), or right arrow for “No” (“No, this face and scene combination was not presented previously”). The A-B pairs were set to always precede the corresponding B-C pair and were separated by a maximum of 10 trials in-between.

Performance was calculated using proportion Hits subtracted by proportion False alarms for both indirect- (i.e., $\frac{c\,o\,r\,r\,e\,c\,t\,r\,e\,s\,p\,o\,n\,s\,e\,s_{\left(t\,r\,u\,e\,s\,c\,e\,n\,e-s\,c\,e\,n\,e\,p\,a\,i\,r\,s\right)}}{t\,o\,t\,a\,l\,t\,r\,i\,a\,l\,s_{\left(t\,r\,u\,e\,s\,c\,e\,n\,e-s\,c\,e\,n\,e\,p\,a\,i\,r\,s\right)}}-\frac{i\,n\,c\,o\,r\,r\,e\,c\,t\,r\,e\,s\,p\,o\,n\,s\,e\,s_{\left(l\,u\,r\,e\,s\,c\,e\,n\,e-s\,c\,e\,n\,e\,p\,a\,i\,r\,s\right)}}{t\,o\,t\,a\,l\,t\,r\,i\,a\,l\,s_{\left(l\,u\,r\,e\,s\,c\,e\,n\,e-s\,c\,e\,n\,e\,p\,a\,i\,r\,s\right)}})$ and direct pair performance (Snodgrass and Corwin, 1988). Unlike most previous studies, we did not require the direct pairs (A-B and B-C) to be correctly remembered for a correctly identified A-C pair to be considered a Hit. This is because we reasoned that memory integration likely is an automatic and largely implicit process, as hypothesized by similar research in the field (Varga et al., 2019). Indirect pairs may as a consequence be correctly endorsed as associated even in the absence of explicit demonstration of recall of the direct pairs underlying the association. Thus, we did not want to limit memory integration to explicit or conscious associative inferences. Task accuracy was extracted by dividing all correct responses with the total amount of trials $\left(A\,c\,c\,u\,r\,a\,c\,y=\frac{c\,o\,r\,r\,e\,c\,t\,r\,e\,s\,p\,o\,n\,s\,e\,s}{t\,o\,t\,a\,l\,t\,r\,i\,a\,l\,s}\right)$. A relative integration score was derived by dividing the accuracy from the indirect pair test with the accuracy from the direct pair test $(\frac{c\,o\,r\,r\,e\,c\,t\,r\,e\,s\,p\,o\,n\,s\,e\,s_{\left(a\,l\,l\,s\,c\,e\,n\,e-s\,c\,e\,n\,e\,p\,a\,i\,r\,s\right)}}{t\,o\,t\,a\,l\,t\,r\,i\,a\,l\,s_{\left(a\,l\,l\,s\,c\,e\,n\,e-s\,c\,e\,n\,e\,p\,a\,i\,r\,s\right)}}/\frac{c\,o\,r\,r\,e\,c\,t\,r\,e\,s\,p\,o\,n\,s\,e\,s_{\left(a\,l\,l\,f\,a\,c\,e-s\,c\,e\,n\,e\,p\,a\,i\,r\,s\right)}}{t\,o\,t\,a\,l\,t\,r\,i\,a\,l\,s_{\left(a\,l\,l\,f\,a\,c\,e-s\,c\,e\,n\,e\,p\,a\,i\,r\,s\right)}}$), as a proxy to estimate the rate of successful integration given each person’s direct (explicit) associative memory capacity. We reasoned that this measure would isolate individual differences in the integration process better than the standard measure used for integration. Specifically, accuracies for indirect pairs reflect a combination of integration and direct associative memory (i.e., memory for directly associated A-B and B-C pairs) because the more direct pairs a person remembers, the more indirect pairs he or she will have the potential correctly endorse or reject based on logical recombination of information from the separate direct pairs at the integration (A-C) test (see Yu et al., 2020). Our relative integration measure also allows estimating integration separately from potential age-differences in direct associative memory. We also report the more conventional integration measure, hit rate adjusted for false-alarm rate, for comparison.

#### Mnemonic Similarity Task

This task was an adaptation of the MST from Stark et al. (2013) and comprised of two parts, a continuous recognition test and a delayed 2-choice forced recognition test, see Supplementary Figure 1B. High quality images were selected for this task from a pool of six premade image sets, taken from publicly available material^3^.

The first part used 152 images shown separately in a continuous pseudorandomized order. The images were on display for 3.5 s followed by a 1.5 s ISI. For each image, the participant had to answer with arrows on a keyboard whether the image currently in display was; a new image not previously seen (foil; left arrow), an old identical image previously seen within the test (target; down arrow), or a similar but not identical image previously seen (lures; right arrow). Non-responses were treated as incorrect answers. Roughly two thirds (110) of the presented images were foils, 40 were targets, and 42 were lures, a total of 192 trials. Lures were distributed in at a range between 3 and 44 trials after the corresponding foil trial, having in total one lure at each distance (42 lures). An appropriate level of difficulty of the critical lure stimuli was determined through piloting, with the final stimuli set comprising of six trials with the most difficult lure bin (lure-bin 1), seven trials with lure-bin 2, seven trials with lure-bin 3, eight trials with lure-bin 4, and 12 trials with lure-bin 5. Discrimination performance was calculated using the Lure Discrimination Index (LDI; Stark et al., 2013, 2015), by taking the difference between the rate of “Similar” responses given to the lure items minus “Similar” responses given to the foil items,$\frac{c\,o\,r\,r\,e\,c\,t\,r\,e\,s\,p\,o\,n\,s\,e\,s_{\left(S\,i\,m\,i\,l\,a\,r\,t\,r\,i\,a\,l\,s\right)}}{t\,o\,t\,a\,l\,t\,r\,i\,a\,l\,s_{\left(S\,i\,m\,i\,l\,a\,r\,t\,r\,i\,a\,l\,s\right)}}-\frac{i\,n\,c\,o\,r\,r\,e\,c\,t\,"\,S\,i\,m\,i\,l\,a\,r\,"\,r\,e\,s\,p\,o\,n\,s\,e\,s_{\left(N\,e\,w\,t\,r\,i\,a\,l\,s\right)}}{t\,o\,t\,a\,l\,t\,r\,i\,a\,l\,s_{\left(N\,e\,w\,t\,r\,i\,a\,l\,s\right)}}$.

The delayed recognition test was administered directly following the continuous recognition paradigm, and comprised 40 images that had been foils and targets in the previous part, but now paired with new lures side-by-side, a total of 40 trials. Participants responded on a keyboard “left arrow” or “right arrow” for which of the two images on display they recognized from the first part of the test. The order and the positioning of the images were pseudorandomized, and each pair was on display for 5 s followed by a 1 s ISI. This test took approximately 2 min. Discrimination performance was evaluated by calculating percent accuracy for correctly chosen objects.

#### Vocabulary Assessment Task

Participants also performed a 30-word vocabulary test (Dureman, 1960; Nilsson et al., 1997). Participants were asked to find a synonym among five possible alternatives for each word. Participants responded by pressing 1–5 on the keyboard, with each number corresponding to an alternative synonym presented on the screen. There was no timeout set for any trial, but participants were informed to respond as quickly as possible. The task included four practice trials with guiding instructions in the beginning. A new trial started immediately after the participant had made a choice, until all 30 trials were finished. The time needed to finish the task differed between participants, ranging from 2 to 7 min. Task performance was measured by the number of correct answers divided with the total of 30 trials ($\frac{c\,o\,r\,r\,e\,c\,t\,r\,e\,s\,p\,o\,n\,s\,e\,s}{t\,o\,t\,a\,l\,t\,r\,i\,a\,l\,s}$).

#### Verbal Fluency

Verbal fluency was assessed in four separate conditions, where participants were required to verbally generate as many words as possible for 1 min according to the following instructions: (1) Words beginning with the letter “A,” (2) Words beginning with the letter “M,” having exactly five letters, (3) Occupations beginning with the letter “B,” (4) Animals beginning with any letter. During scoring every generated word was checked against the Swedish Academy Dictionary (SAOB), the Swedish Academy Glossary (SAOL), and a Swedish Dictionary (SO). Slang words and known variants were approved as words, whereas English (or other languages) versions of words or occupations were not. Repeated words and inflected versions of the same word (dove – doves) were not approved. Similarly, words describing the same type or kind (lamb – sheep) were not approved for the animal fluency condition. The outcome measure for verbal fluency was a composite score, calculated by summing all valid responses across the four separate conditions.

#### Perceptual Processing Speed

Perceptual speed was assessed with two tasks; a letter-comparison task and a figure-comparison task (Schmiedek et al., 2010; Nevalainen et al., 2015). Participants were asked to distinguish between two stimuli presented side by side, with aim to determine as quickly as possible if the stimuli matched or not. Participants responded with the left arrow on their keyboard for ‘‘yes, the sequences/figures seen on the screen are identical’’ or the right arrow for ‘‘no, the sequences/figures seen are not identical.’’ The letter-comparison stimuli were four-letter strings (a-z), where the letters in both strings were the same (identical), or differed by one letter in either string (not identical). The figure-comparison were two figures (‘‘fribbles’’; courtesy of Michael J. Tarr, Brown University, Providence, RI, United States)^4^ presented close to each other, with either both figures looking the same (identical), or differed in one component (not identical). The trial ended with either a button press or automatically after 5 s (timeout). The ISI between a response or timeout, and appearance of a new item was 0.5 s. Each test consisted of 40 item pairs, of which half were identical and intermixed with the other half of differing pairs (Nevalainen et al., 2015). One practice run with 40 items was presented before the two test trials for each task (e.g., letter condition: practice → test1 → test2). The total score was summed for each of the two tests separately by dividing the number of correct responses by the total response time (i.e., for both correct and incorrect responses; in milliseconds) and multiplying this quotient by 60,000 ($\frac{c\,o\,r\,r\,e\,c\,t\,r\,e\,s\,p\,o\,n\,s\,e\,s}{t\,o\,t\,a\,l\,r\,e\,s\,p\,o\,n\,s\,e\,t\,i\,m\,e}\times 60 000)$, i.e., creating a score of correct responses per minute, while also penalizing incorrect responses.

### Statistical Procedures

Prior to statistical analyses, all outcome measures were Z-standardized to a mean of 0 and standard deviation of 1. Response times (RTs) were median values summarized from all trials (both correct and incorrect responses), to avoid influence of extreme values as the RT distribution was positively skewed. Missing responses were included in the total amount of trials for accuracy analyses. Participants with missing data, and mean values beyond 3 standard deviations from the age-group mean were considered outliers for that task only, and removed prior to analysis. The number of included participants thus differ depending on the task (see Table 1). In sum, for the MST delayed recognition task, three participants were removed for being outliers; for the fluency task, three participants were excluded due to technical problems; for perceptual speed, two participants were excluded due to technical problems; for the AIT, one participant was removed for having missing responses on more than half of the trials.

**TABLE 1:** Task performance by age group.

	Younger adults			Middle-aged adults		
Cognitive domain (performance)	*M*	SD	*n*	*M*	SD	*n*
**Memory integration, AIT**						
*Indirect retrieval (Hits-FA)*	0.41	0.25	41	0.31	0.22	39
*Indirect retrieval (FA)*	0.24	0.15	41	0.28	0.16	39
*Direct retrieval (Hits-FA)*	0.58	0.23	41	0.46	0.23	39
*Direct retrieval (FA)*	0.17	0.14	41	0.22	0.16	39
*Relative integration score*	0.82	0.14	41	0.83	0.21	39
**Memory discrimination, MST**						
*Continuous (% accuracy)*	86.0	5.0	41	86.1	4.2	40
*New (% accuracy)*	94.5	5.5	41	95.5	3.5	40
*Old (% accuracy)*	81.5	11.3	41	78.2	12.1	40
*Similar (% accuracy)*	67.9	17.3	41	69.2	12.2	40
*LDI (relative score)*	63.9	16.5	41	65.9	12.7	40
*Delayed recognition (% accuracy)*	90.9	5.2	40	88.2	7.6	38
**Verbal semantic memory**						
*Vocabulary (% accuracy)*	64.6	14.4	41	76.3	13.6	40
*Verbal fluency (composite score)*	44.7	11.8	39	53.5	14.6	39
**Perceptual speed**						
*Fribbles (score)*	22.8	4.2	39	19.3	3.9	40
*Letters (score)*	45.7	10.7	39	38.7	10.0	40

*M, Mean value; SD, Standard deviation; Hits-FA, proportion Hits minus proportion False Alarms; FA, proportion False Alarms; AIT, Associative inference task; MST, Mnemonic similarity task; LDI, Lure discrimination index. For calculation of the outcome measures, please refer to the “Materials and Methods” section.*

We investigated the effect of age-group on all outcome measures using a linear regression model with an indicator variable for age-group (0 = young adults; 1 = middle-aged), along with sex and years of education as covariates of no interest. Hypothesis tests were done using Bayes factors (Kass and Raftery, 1995) as indices of relative evidence of one statistical model over another, while also allowing a basis for statistical support favoring a null hypothesis (Heck et al., 2021).

The same prior was used for all parameters in the models, and was selected to be normally distributed with a mean of 0 and a standard deviation of 1, i.e., *Normal*(0, 1). As all outcome measures were z-transformed, the *N*(0, 1) prior is weakly informative, favoring smaller effects but not excluding effects of a magnitude comparable to the variation in the data. Age-effects from episodic memory-, vocabulary-, and perceptual speed tasks measures are unlikely to show large effect sizes for comparisons between younger- and middle-aged adults. Standardized effect sizes ranging from 0.5 to 1 have been reported for these measures when comparing middle-aged with young adults (Schaie, 1989; Salthouse, 1996; Stark et al., 2013; Cansino et al., 2018).

The null hypothesis (H_0_) was the same in every regression model and was defined by a null region, instead of the more common point-null. The reason being that a point-null can be viewed as unrealistic, as it would require the investigated effect to be exactly zero (Heck et al., 2021). The null region included effect sizes ranging from −0.1 to 0.1 standard deviation units from 0. This cut off was chosen as half of the commonly used definition of a small effect size, i.e., 0.2 (Cohen, 1988). Any effect within this null region was thus considered too small to be theoretically relevant. As a control analysis for findings on memory integration and discrimination, we repeated the analyses using a point-null hypothesis, which revealed that the pattern of results remained unchanged, although the magnitude of the BFs for null were slightly attenuated.

Statistical analyses were conducted in R software v.4.0.5 (R Core Team, 2018). BFs were calculated using bayestestR package v0.9.0 (Makowski et al., 2019), which is a compiled package consisting of rstanarm (Goodrich et al., 2020) and brms (Bürkner, 2017), amongst other R packages.

## Results

### Middle-Aged Adults Perform Better on Verbal Knowledge

First, we tested whether the middle-age group had an advantage in verbal knowledge, as predicted by the information accumulation hypothesis. We investigated the effect of age-group on the vocabulary test and verbal fluency composite score using linear regression models with an age-group indicator variable, along with sex and years of education. As stated previously, we performed two-sided tests for the age effects (H_1_), using a null region centered around zero (H_0_). The BF for the vocabulary task showed support for H_1_ being 39.8 times more likely than H_0_, with a posterior distribution mean of 0.71 (Table 2; 95% CI: 0.29, 1.12). Similarly, data for word fluency showed support for H_1_ being 8.5 times more likely than H_0_ (Table 2; posterior mean = 0.61; 95% CI: 0.17, 1.04). The BF values and the direction of the posterior distribution for both vocabulary tests indicate that the middle-aged adults are around 8–40 times more probable to perform better than younger adults, to the degree of at least 0.1 standard deviations from a zero difference. Thus, in line with previous literature in the field, the results show that given the data, middle-aged adults are more probable to have better verbal knowledge than younger adults (see also Tables 1, 3 for descriptives).

**TABLE 2:** Bayes factor values and posterior distributions (z-scores) for the effect of age-group, from separate regression models for each task, adjusting for sex and education.

		95% Credible interval		
Task	pMean	Lower	Upper	BF_10_
**AIT (integration)**				
*Direct pairs (Hits-FA)*	–0.54	–0.99	–0.09	3.17
*Indirect pairs (Hits-FA)*	–0.39	–0.84	0.07	0.80
*Relative integration score*	0.10	–0.37	0.56	0.19
**MST (discrimination)**				
*LDI*	0.17	–0.30	0.63	0.24
*Delayed recognition*	–0.18	–0.64	0.29	0.25
**Verbal knowledge**				
*Vocabulary*	0.71	0.29	1.12	39.77
*Verbal fluency composite*	0.61	0.17	1.04	8.52
**Perceptual speed**				
*Fribbles*	–0.76	–1.17	–0.35	73.52
*Letters*	–0.62	–1.03	–0.20	11.43

*pMean, posterior mean value (z-score; positive value = performance difference in favor of middle-aged adults; negative value = in favor of younger adults); BF_10_, Bayes factor in favor of the alternative hypothesis (an age-group difference in any direction); AIT, Associative inference task; MST, Mnemonic similarity task; Hits-FA, proportion Hits minus proportion False Alarms; LDI, Lure discrimination index.*

**TABLE 3:** Task response times in milliseconds by age group.

	Younger adults		Middle-aged adults	
Task	M	SD	M	SD
**Memory integration, AIT**				
*Indirect retrieval*	2285	489	2620	496
*Direct retrieval*	1771	343	2090	482
**Memory discrimination, MST**				
*Continuous (all trials)*	1193	216	1329	255
*New*	1051	285	1168	284
*Old*	1366	266	1579	347
*Similar*	1402	255	1551	256
*Delayed recognition*	1371	291	1484	316
**Verbal semantic memory**				
*Vocabulary*	7328	1667	6387	2249

*M, Mean value; SD, Standard deviation; AIT, Associative inference task; MST, Mnemonic similarity task, response times reflect both correct and incorrect responses.*

### No Effect of Age-Group on Memory Integration Ability

**FIGURE 1:**
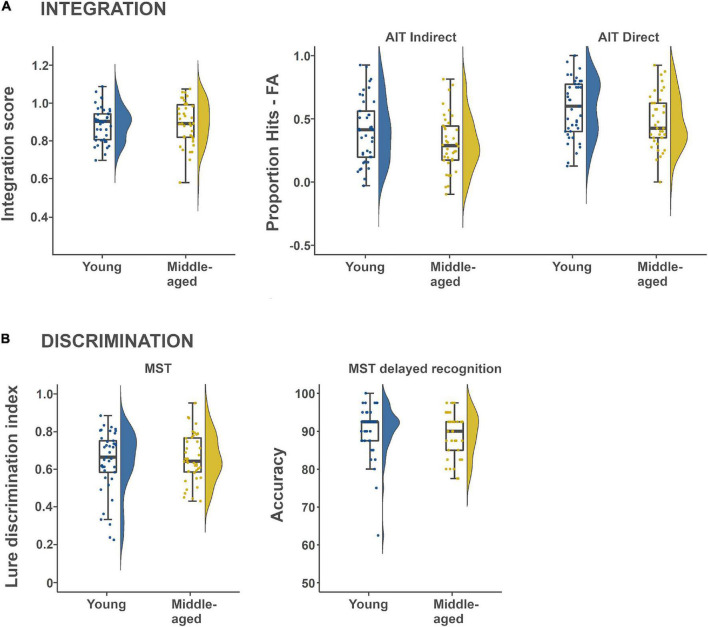
Descriptive box plots and violin plots showing task performance distribution within and between age-groups. **(A)** Shows memory intergration performance derived from the Associative Inference Task (AIT), while **(B)** shows mnemonic discrimination performance derived from the Mnemonic Similarity Task (MST).

Two different measures were extracted from the same task, the more conventional Hits-False alarm rate and a relative integration score, where the latter intended to capture integration differences over and above direct associative memory. We first tested if the A-C (scene-scene) pair task performance was above chance for each age-group separately, using a Bayesian one-sample *t*-test with Hits-FA as the outcome variable, where the difference was assigned a uniform prior on (−1, 1). For both age-groups (Table 1; Younger adults: Posterior mean = 0.41, Posterior SD = 0.25; Middle-aged adults: Posterior mean = 0.31, Posterior SD = 0.22), the analyses show that it is more than 1000 times likely (BFs > 1000) that performance was better than chance. Secondly, we tested for an age-effect on the integration score, which was a relative rate of successfully integrated A-C pairings, given rate of successfully retrieved A-B and B-C pairs. The BF in favor of the null region was 1/0.192 = 5.3 (Table 2), with a posterior mean of −0.10 for the age effect, and a 95% CI is given by (−0.37, 0.56) indicating that the effect of age-group is around five times more probable to be of too small magnitude to be considered relevant (see Tables 1, 3 and Figure 1A for descriptives). In a similar fashion, we lastly investigated an effect of age-group on A-C pair performance (Hits-FA). The BF in favor of the null region was 1/0.806 = 1.24 (Table 2), showing that H_1_ and H_0_ predict the data equally well. Here, the posterior mean of −0.39, and a 95% CI of the group effect is given by (−0.84, 0.07), see Figure 1A and Tables 1, 3 for descriptives. Thus, the integration score data were most consistent with there being no meaningful difference between young- and middle-aged adults in memory integration ability, while the indirect pair performance data did not provide conclusive evidence for the absence or presence of an age-effect.

### Evidence for Younger Adults Having Higher Associative Inference Task Direct-Pair Performance

A similar analysis was set up for the direct face-scene retrieval performance to test for an age-difference in remembering the direct pairs. The BF was 3.2, showing that H_1_ is three times more likely than H_0_ given the data (Table 2). The corresponding posterior mean was −0.54 and a 95% CI of the group effect is given by (−0.99, 0.09). The direction of the posterior further indicates that younger adults were more probable to perform better on face-scene retrieval (see also Figure 1A and Tables 1, 3), for descriptives.

### Middle-Aged Adults Show Similar Memory Discrimination Ability as Younger Adults

Our second main hypothesis of a decreased ability to discriminate details in episodic memories in midlife was tested with continuous- and delayed recognition data from the MST. The analyses were set up as previous regression models with first the LDI score (see section “Materials and Methods” and Stark et al., 2013) from the MST continuous recognition condition. The BF in favor of the null was 1/0.241 = 4.2, with an age-effect posterior mean of 0.17 (Table 2; 95% CI: −0.30, 0.63). Next, for delayed recognition performance, the BF in favor of the null was 1/0.251 = 4.0, and the corresponding posterior mean was −0.18 (95% CI: −0.64, 0.29). Descriptives are shown in Figure 1B and Tables 1, 3. Thus, contrary to our hypothesis, the effect of age-group on discrimination performance was around 4 times more probable to be of negligible magnitude.

### No Evidence for an Association Between Integration and Discrimination Performance

We also tested whether the sometimes reported trade-off between integration ability and discrimination could be observed across tasks in our sample. To do so, separate models were set up with integration score as the dependent variable, and the predictor of interest (either LDI or delayed recognition accuracy). Interaction terms between the discrimination variable and age group were also tested in separate models to allow for differential associations across age-groups. In these regression models, the H_0_ null region was set to (−0.1, 0.1) and tested against H_1_ where discrimination performance, or an age-by-discrimination interaction, could explain integration ability. The BF for the interaction term in both regression models was estimated to be in favor of H_0_, by 1/0.238 = 4.2 times and 1/0.245 = 4.1 times, respectively, showing evidence for negligible interaction effects between discrimination performance and age-group. In other words, the association between integration and discrimination performance did not differ between the age-groups. In the regression model with LDI as a predictor of integration performance, the BF in favor of H_0_ was 1/0.058 = 17.2, with a posterior mean of −0.03 (95% CI: −0.26, 0.20). In the regression model with delayed recognition as a predictor, the obtained BF in favor of H_0_ was 1/0.059 = 16.9, with a posterior mean of −0.03 (95% CI: −0.26, 0.20). Thus, any relationship between integration and discrimination, as measured in this study, was around 17 times more probable to be of negligible magnitude.

### Middle-Aged Adults Have Generally Slower Response Times and Perceptual Speed

As an additional analysis for the main memory tasks, we tested whether response times (RTs) differed between age-groups. Descriptives can be seen in Table 3. Separate regression models were set up with RTs for each task as the dependent variable and age-group as the predictor of interest. Across all task variables, the BFs (3.3–35.1; see Table 4) and posterior distributions showed evidence for middle-aged adults having longer RTs, with the exception of “New” trials in the MST continuous paradigm, and for delayed recognition, which both showed inconclusive evidence (BF_10_ = 0.76 and 0.77, respectively; Table 4), likely due to ceiling effects in accuracy (Table 1). In addition, no evidence was obtained for speed-accuracy trade-offs, or age-differences in the RT-accuracy relationships, for any memory variable (see Supplementary Material text for analysis details and Supplementary Table 2 for results). Finally, we also investigated age-effects in perceptual speed assessed by a separate task. For the condition involving pictorial stimuli (outcome measure: correct responses per minute), the BF in favor of H_1_ was 73.5, with a posterior distribution mean of −0.76 (Table 2; 95% CI: −1.17, −0.35). Similarly, the BF for the letter comparison processing speed condition (correct responses per minute) showed support for H_1_ being 11.4 times more likely than H_0_, with a posterior mean of −0.62 (95% CI: −1.03, −0.20). The directions of the posterior in both conditions show that the younger adults were more probable to perform faster than 0.1 standard deviations from no group difference, and more convincingly so for the pictorial stimuli (see Table 1 for descriptives). Thus, in accordance with prior literature, our middle-aged sample showed a general slowing across both RTs and perceptual speed task performance.

**TABLE 4:** Bayes factor values and posterior distributions (z-scores) for the effect of age-group on response times, from separate regression models adjusting for sex and education.

		95% Credible interval		
Task	pMean	Lower	Upper	BF_10_
**AIT Response times**				
*Indirect retrieval*	0.54	0.10	0.97	4.13
*Direct retrieval*	0.71	0.29	1.13	35.13
**MST Response times**				
*Continuous (all trial types)*	0.52	0.08	0.94	3.30
*New*	0.36	–0.08	0.80	0.76
*Old*	0.61	0.19	1.04	9.19
*Similar*	0.57	0.13	1.01	5.20
*Delayed recognition*	0.36	–0.09	0.81	0.77

*pMean, posterior mean value (z-score; positive value = performance difference in favor of middle-aged adults; negative value = in favor of younger adults); BF_10_, Bayes factor in favor of the alternative hypothesis (an age-group difference in any direction); AIT, Associative inference task; MST, Mnemonic similarity task.*

## Discussion

The present study set out to investigate the possibility that healthy middle-aged adults would show an advantage in integration ability, combined with a reduction in discrimination ability. Contrary to hypotheses, the data showed evidence for middle-aged adults performing similarly to young adults on tasks designed to capture mnemonic integration of indirectly related stimulus pairs, and discrimination of details from episodic memory. Thus, no advantageous differences in favor for middle-aged adults were observed beyond what can be expected in midlife, i.e., better verbal knowledge. Instead, a disadvantage in for the middle-aged group could be observed on the direct face-scene pairs of the associative inference memory task. The middle-aged also showed an expected slowing of perceptual speed. Thus, the age-equivalence in integration and discrimination abilities were observed in the presence of expected age-related differences in other cognitive tasks, demonstrating that they were unlikely driven exclusively by sample selectivity. In the discussion to follow, we consider our results in light of the current literature, and consider possible reasons behind observed age-group differences or the lack thereof.

Based on prior research showing that memory integration can be one effective way to handle memory interference (e.g., Postman, 1964; Anderson and McCulloch, 1999), we hypothesized that an improvement in memory integration may be seen in healthy middle-aged adults when compared to young. Our main measure of integration instead indicated that performance was age-equivalent, whereas the data for the indirect pair performance (another proxy for integration) were inconclusive. There could be several reasons for not observing the hypothesized age-advantage. First, despite the oftentimes highlighted adaptiveness of human memory and cognition (Anderson and Milson, 1989; Woolgar et al., 2015), it is of course possible that adaptations such as relative increases in certain memory sub-processes in response to changes in internal operating conditions, as those arising from increased interference from accumulated memory traces across life, simply do not occur. Instead, major memory impairment due to interference in everyday life could be avoided trough sufficiently functioning neuro-computational pattern separation (Hunsaker and Kesner, 2013) throughout most of adulthood. Other means to prevent interference could be, for instance increased adaptive forgetting (Richards and Frankland, 2017) or utilizing changes in temporal context to differentiate memories (El-Kalliny et al., 2019). That would not preclude observing interference from specific prior knowledge in certain contexts, as predicted by the information accumulation perspective of cognitive aging (Ramscar et al., 2014; Qiu and Johns, 2020). It is also possible that at least some of the real-life memory integration relevant for interference-reduction happens during offline consolidation (Tompary and Davachi, 2017), and thus was not captured by our testing procedures.

Another potential reason for not observing the hypothesized integration benefit in middle-aged, is that current task procedures may have lacked critical elements for eliciting it. Specifically, one common element in previous studies observing beneficial effects of integration on memory interference-reduction or retention is that most of them capitalized on prior knowledge for eliciting the integrative process (e.g., Anderson and McCulloch, 1999). This can be exemplified by Postman’s (1964) use of semantically related words to bridge across overlapping consecutive item lists to be learned. Since our version of the AIT involved only arbitrary associations between novel stimuli, it is possible that a benefit on memory integration in midlife would be observed in task paradigms enabling middle-aged adults to use their more extensive general world knowledge to their advantage (cf., Ryan et al., 2020). After all, in adulthood real-life memory integration, as with memory and learning in general, rarely happens void of past experiences, but is embedded in a prior knowledge context (Umanath and Marsh, 2014; Brod and Shing, 2019; Spreng and Turner, 2019). Thus, it is possible that the more extensive real-life experience of integrating new information into pre-existing knowledge among middle-aged would translate to a performance benefit only in more ecologically valid tasks where pre-existing knowledge could be used to support the integration process. The AIT, while being a widely used test of integration (Preston et al., 2004; Schlichting et al., 2015), also has another potential drawback in that high performance on the indirect (i.e., integration) test may be achieved by means of high recall of the direct A-B and B-C pairs. Specifically, recalling both the directly studied pairs underlying an A-C stimulus pair allows endorsement or rejection of that stimulus pair without necessarily evoking an integrative process (Kumaran and McClelland, 2012; Yu et al., 2020). Although neuroimaging provides evidence for integration being evoked in AIT (e.g., Shohamy and Wagner, 2008; Schlichting et al., 2014), behavioral measures alone cannot adequately differentiate high performance due to integrative processing, from high performance by means of high direct associative memory (although see Yu et al., 2020). Our relative integration score was intended to capture integrative ability over and above that expected from generally high associative memory. Despite these efforts, no hypothesized age-differences could be reliably detected on the relative integration score, beyond what is considered a very low effect size of 0.1 standard deviations. Nevertheless, the current results contribute to the literature on memory processing across the lifespan in showing that memory integration, as captured by the AIT, does not necessarily show a negative age-effect in healthy middle-aged adults, but rather, that they can perform seemingly on par with younger adults. This is particularly noteworthy given the lower performance of the middle-aged for the direct pairs in the AIT, as discussed next. Tentatively, this may indicate a relative sparing of integration over some other memory processes in midlife.

Somewhat unexpectedly for our relatively young and healthy middle-aged sample, we found evidence for a negative age-difference in retrieval of directly studied associations. Some of the current literature suggests that general associative memory decrement can be detected cross-sectionally in middle-aged compared to young adults (e.g., de Chastelaine et al., 2016) or observed as a linear decline across the lifespan (Henson et al., 2016), which could be in line with the associative deficit hypothesis of cognitive aging (Bastin and Van der Linden, 2006; Naveh-Benjamin and Mayr, 2018). Other studies have observed negative age-effects for healthy middle-aged adults on context and source memory tests (Kwon et al., 2016; Cansino et al., 2018), which also require associative binding of stimuli with their sources and contexts. The current results could thus be interpreted as in line with a possible age-difference between young and middle-aged in the ability to remember relationships between unrelated stimuli. If this is true, it is of relevance to consider whether the preserved integration observed in the middle-aged involves a different binding process than the direct associations between stimulus pairs. As far as we are aware, this is still an empirical question, although some theoretical models posit the existence of different forms of biding underlying memory, separating within-episode binding from across episode-binding (Opitz, 2010), and explicit from implicit associative binding (Davis et al., 2021). On the other hand, from the present observations we cannot distinguish whether the lower performance of the middle-aged group on the direct pairs of the AIT was driven by an associative binding deficit in explicit memory, or by differential handling of the pro- and retroactive interference that is built in to the AIT by the overlapping A-B and B-C stimulus pairs. Nevertheless, our results suggest that future studies aimed at finding sensitive measures of early age-related declines in middle-age may benefit from further examining associative memory measures, with and without interference, rather than tests involving memory integration or discrimination.

Also contrary to our hypothesis, the data from MST showed robust absence of negative age-effects on two different measures of memory discrimination. This is in contrast to previous studies that investigated discrimination in middle-aged individuals (Stark et al., 2013; Nauer et al., 2020; Güsten et al., 2021). The discrepancy may be due to the fact that our middle-aged sample was relatively young and screened for health conditions known to affect cognition, and therefore healthier than the participants in previous studies. Alternatively, the continuous recognition setup of our task with shorter retention intervals may have rendered the task easier, perhaps in combination with the more generous responding times employed in our task (3.5 vs. 2 s). In light of the commonly observed slower perceptual processing speed in middle-aged compared to young adults, the more generous responding times may have reduced age-effects related to processing speed or task stress. Yet, the overall LDI performance was not close to ceiling (see Figure 1B), even in our young participants, which indicates that the task difficulty was appropriate, and that it would have been possible to capture age-effects if such existed in the sample. In sum, although our results as cannot be taken to establish that a discrimination deficit is never present in middle-age, they nevertheless positively indicate that discrimination does not necessarily decline to middle-age in healthy adults.

We also note that we did not observe any evidence for negative associations between our integration and discrimination measures, that may have been indicative of a hypothesized and sometimes observed trade-off between these processes (Sweegers and Talamini, 2014; van Kesteren et al., 2016; Tompary and Davachi, 2017). Neither did we observe evidence for differential associations between integration and discrimination in middle-aged compared to younger adults, which could have indicated age-related differences in relative deployment of the two processes. We acknowledge that our tasks were not designed to detect such trade-offs, and that they are likely challenging to capture across tasks and individuals due to the positive manifold of cognitive abilities (Spearman, 1927). Recent evidence indicates that integration and discrimination likely operate independently and in parallel (Banino et al., 2016; Keresztes et al., 2018; Yu et al., 2020), and are sub-served by different neural substrates (Schlichting et al., 2014; Brunec et al., 2020). If so, the possibility remains that experience-related adaptive changes in memory processing or detrimental neurocognitive declines past midlife may affect these processes differentially.

The validity of our results is supported by our replication of well-known performance differences in midlife, i.e., increased verbal knowledge and slower perceptual processing speed. At the same time we acknowledge that our cross-sectional study design is limited for drawing conclusions about aging- or developmental changes. The relatively small age-difference between the young and middle-aged adults should have minimized potential cohort- or generational differences known to cause bias in aging studies (e.g., differential educational attainment; Rönnlund et al., 2005; Lenehan et al., 2015; Kobayashi et al., 2017), but longitudinal designs are nevertheless more sensitive in detecting aging-effects by virtue of measuring the same individuals over time. Further, the use of self-reported health measures to screen our sample may have missed some cases of health conditions that are more common in midlife (e.g., hypertension or type-2 diabetes), affecting the neurocognitive integrity and cognitive performance of the middle-aged group negatively. Inclusion of neuroimaging measures could have revealed such subtle age-related neurodegenerative changes, as has previously been demonstrated in middle-aged adults (Elobeid et al., 2012; Ferreira et al., 2017; Elliott et al., 2019). Finally, our online testing format also entailed less control over the test situation, including potential noise caused by for instance screen resolution and size, connection quality, and surrounding environment. However, prescreening procedures ensured that participants had suitable equipment, and having the test-leader contacting each participant over a video call mitigated concerns about test environment, task understanding, and compliance with instructions. Thus, the validity of the data should not have been compromised by the online testing format, as also evidenced by the ability of our data to capture some known age-differences expected from the literature.

## Conclusion

Although the main hypothesis of a memory integration advantage in midlife was not supported, our data provide evidence demonstrating that healthy middle-aged adults can be as proficient as younger adults in both memory integration and discrimination. Thus, despite a general slowing of processing speed, age-related deficits in these core processes underlying episodic memory do not appear to be necessary consequences of reaching midlife. These findings challenge the generality of previously observed discrimination deficits in midlife, and contribute new knowledge regarding the potential for preserved memory integration up to midlife in healthy adults. Future longitudinal studies are needed to substantiate the current cross-sectional observations, in addition to systematic investigation of adverse health parameters potentially affecting their generality. We believe that investigation of task conditions that allow middle-aged individuals to use their more extensive semantic knowledge to support memory processes such as integration constitutes a more ecologically valid and fruitful future avenue for investigating beneficial adaptive changes in memory processes across the lifespan.

## Data Availability Statement

The raw data supporting the conclusions of this article will be made available by the authors, without undue reservation.

## Ethics Statement

This study involving human participants was reviewed and approved by the Swedish Ethical Review Authority. Written informed consent was not provided because the study was conducted online and participants provided verbal informed consent after having read the study information. Informed consent statements were audio recorded. The procedure was approved by the Ethics Review Authority. The individual(s) provided their written informed consent for the publication of any identifiable images or data presented in this article.

## Author Contributions

GS: data curation, visualization, software, formal analysis, writing – original draft, and writing – review and editing. AL: supervision and writing – review and editing. SP: conceptualization, funding acquisition, project administration, supervision, writing – original draft, and writing – review and editing. All authors contributed to the article and approved the submitted version.

## Conflict of Interest

The authors declare that the research was conducted in the absence of any commercial or financial relationships that could be construed as a potential conflict of interest.

## Publisher’s Note

All claims expressed in this article are solely those of the authors and do not necessarily represent those of their affiliated organizations, or those of the publisher, the editors and the reviewers. Any product that may be evaluated in this article, or claim that may be made by its manufacturer, is not guaranteed or endorsed by the publisher.

## Footnotes

1 Here, we do not intend to study forms of integration pertaining to within-stimulus binding of stimulus features (unitization), or within-episode binding of for instance two simultaneously presented stimuli, or a stimulus and its spatio-temporal context.

2
https://pavlovia.org

3
https://github.com/celstark/MST

4
http://www.tarrlab.org
