# Supplementary material for: Healthy Middle-Aged Adults Have Preserved Mnemonic Discrimination and Integration, While Showing No Detectable Memory Benefits
Source: Front Psychol. 2022 Jan 24;12:797387. doi: 10.3389/fpsyg.2021.797387 (PMC8819667; doi:10.3389/fpsyg.2021.797387)
Supplement: Supplementary file 1 [file Data_Sheet_1.pdf]

Supplementary Material

Supplementary Text

Speed-accuracy analysis

To test whether our results were affected or driven by speed accuracy trade-offs, separate regression models were set up with task accuracy as the dependent variable for all memory variables (AIT indirect, AIT direct, MST continuous recognition, MST delayed recognition) and the predictor of interest (RTs from each task respectively). An interaction term between the RT variable and age-group was also included in a separate model to test for differential associations between groups. In all regression models, the H_0 null region for the RT effect and the age-by-RT interaction was set to [-0.1; 0.1] and tested against $H_{1;}$ where RTs is associated with accuracy in any direction, or an age-by-RT interaction was present. Results are given in Supplementary table 2. In sum, BFs in favor of H_0 showed around 1.9 to 16.7 times higher probabilities for negligible speed-accuracy trade-offs, or age-differences in the RT-accuracy relationships, with exception for AIT direct retrieval, where the RT data showed anecdotal evidence (BF_{10}=1.7) for H_1 across the whole sample. The posterior mean (-0.29) indicated that higher accuracy on AIT direct pairs was associated with marginally faster RTs, thus the opposite of a speed-accuracy trade-off.

Supplementary table 1. The total number of excluded individuals during the recruitment process

**Exclusion criteria**	**Details**	**Younger adults (n)**	**Middle-aged adults (n)**
Pharmacological treatment	*Any treatment that may affect cognition*	2	1
Neurological disorders	*e.g. ADHD, head trauma*	4	0
Severe somatic diseases	*e.g. chronical somatic diseases*	1	2
Psychiatric disorders	*e.g. depression*	8	3
Cardiovascular-conditions	*e.g. heart attack, hypertension*	1	8
Diabetes		0	1

Total: 31

Supplementary table 2. Bayes factor values and posterior distributions (z-scores) for the association between median response times and the respective task performance, as well as for age-by-response time-interactions. Age-group, sex and education were always included as covariates in the regression models.

		95% Credible interval		
**Variable**	**pMean**	Lower	Upper	**BF_{10}**
AIT indirect retrieval (Hits-FA)				
Response time	-0.22	-0.45	0.01	0.52
Response time * age-group	-0.17	-0.64	0.29	0.25
AIT direct retrieval (Hits-FA)				
Response time	-0.29	-0.51	-0.06	1.73
Response time * age-group	0.22	-0.27	0.69	0.31
MST continuous (accuracy)				
Response time	-0.26	-0.36	0.10	0.14
Response time * age-group	0.10	-0.36	0.57	0.20
MST delayed recognition (accuracy)				
Response time	-0.01	-0.24	0.24	0.06
Response time * age-group	-0.09	-0.55	0.37	0.19

Note: pMean = posterior mean value (z.score), BF_{10} = Bayes factor in favor of the alternative hypothesis (an association between performance and response times, or an age-by-response time-interaction; which corresponds to the probability of an effect larger than the null region of [-0.1, 0.1] SD), Hits-FA = proportion Hits minus proportion False Alarms, AIT = Associative inference task, MST = Mnemonic similarity task

Supplementary Figures

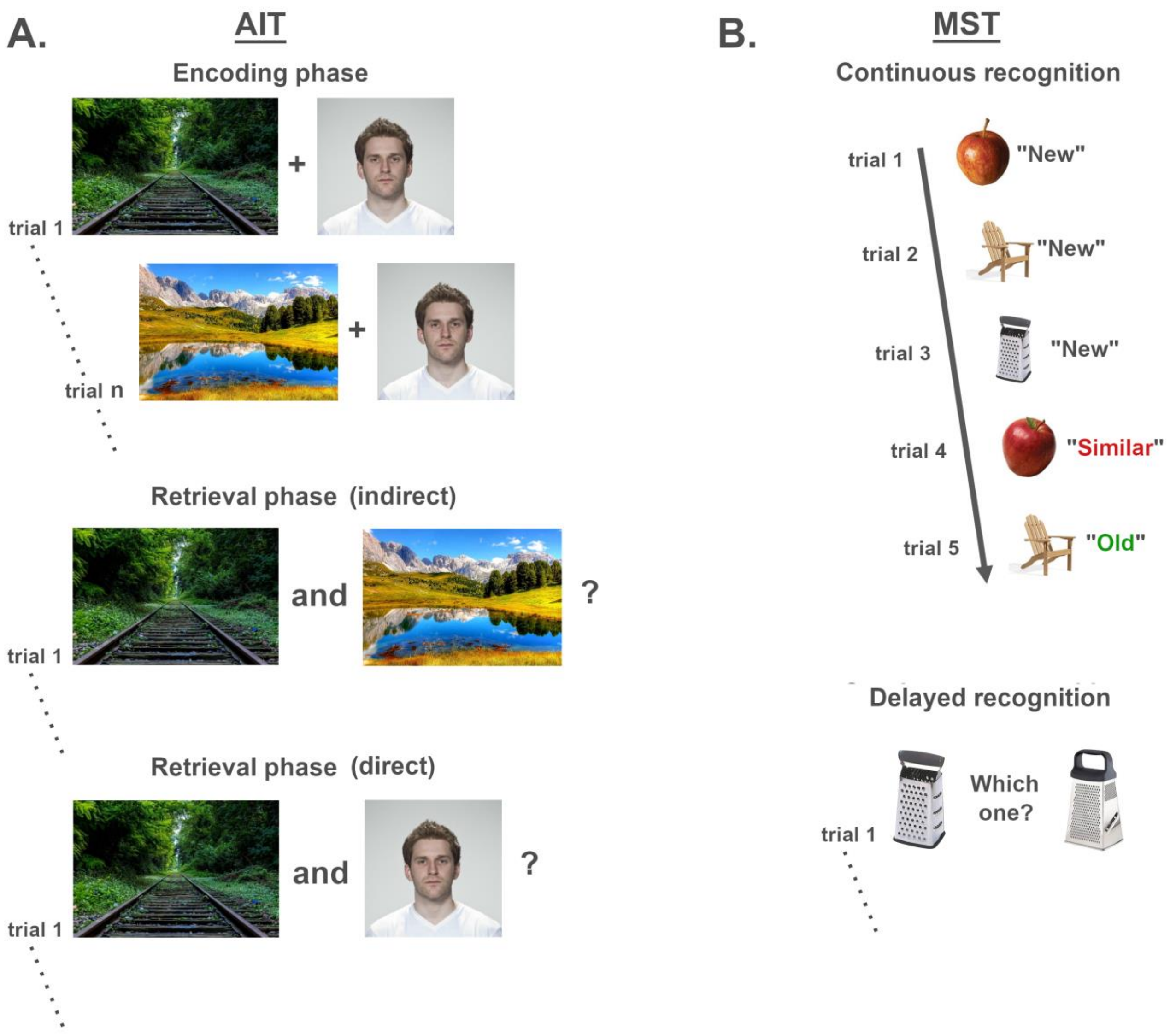

Supplementary figure 1. Task design and trial organization, A. Associative inference task (AIT) divided into three phases. B. Mnemonic similarity task (MST) divided into two phases. The words within quotation marks indicate correct answers, and were not shown to the participants. Source for face photo: DeBruine, L., & Jones, B. (2017). *Face Research Lab London Set* (Version 5). figshare. https://doi.org/10.6084/m9.figshare.5047666.v5. All individuals gave signed consent for their images to be "used in lab-based and web-based studies in their original or altered forms and to illustrate research (e.g., in scientific journals, news media or presentations)".
